# New Method of Treating Urethral Strictures

**Published:** 1877-03

**Authors:** 


New Method of Treating Urethral Strictures.—At the meeting of the *Academic de Medecine* at Paris, on November 7th, M. C. Fort described a method of treating urethral strictures of small calibre, which he has employed for seven years with great success. He first passes a filiform bougie through the stricture and leaves it in position for twenty-four hours. The presence of the bougie excites a slight inflammation of the tissues that form the stricture, which softens them and renders them more extensible. The bougie is provided with a metallic cap, to which catheters of various sizes may be attached. At the end of the twenty-four hours, a conical catheter, three millimetres in diameter at the largest part, is attached to the bougie, and pushed forward through the stricture, the guide keeping it in the proper direction. The catheter is then withdrawn until the metallic cap on the end of the bougie emerges from the center. The catheter is then detached from the bougie and replaced by another, which is five millimetres in diameter at the largest part. This is in turn made to traverse the stricture, then withdrawn, and replaced by a third catheter which measures seven millimeters in its largest diameter. The bougie during the passage of the catheters coils up in the bladder, where it can do no harm.

By this method even very tight and indurated strictures may be completely dilated at a single sitting. The guide removes all danger of making a false passage. The pain is so slight that anæsthesia is unnecessary. The operation rarely causes any hemorrhage. During the seven years that M. C. Fort has employed this method, he has never known it to be followed by any complications except an occasional attack of urethral fever, which has always readily yielded to quinine. To prevent a relapse, a catheter must be passed at intervals for a long time.—*Gazetta Medicale de Paris*, November 11, 1876.

